# Ocular Inserts for Sustained Release of the Angiotensin-Converting Enzyme 2 Activator, Diminazene Aceturate, to Treat Glaucoma in Rats

**DOI:** 10.1371/journal.pone.0133149

**Published:** 2015-07-23

**Authors:** Giselle Foureaux, Juçara Ribeiro Franca, José Carlos Nogueira, Gustavo de Oliveira Fulgêncio, Tatiana Gomes Ribeiro, Rachel Oliveira Castilho, Maria Irene Yoshida, Leonardo Lima Fuscaldi, Simone Odília Antunes Fernandes, Valbert Nascimento Cardoso, Sebastião Cronemberger, André Augusto Gomes Faraco, Anderson José Ferreira

**Affiliations:** 1 Department of Morphology, Federal University of Minas Gerais, Belo Horizonte, Minas Gerais, Brazil; 2 Department of Pharmaceutical Products, Federal University of Minas Gerais, Belo Horizonte, Minas Gerais, Brazil; 3 Institute of Exact Sciences, Federal University of Minas Gerais, Belo Horizonte, Minas Gerais, Brazil; 4 Department of Clinical and Toxicological Analysis, Federal University of Minas Gerais, Belo Horizonte, Minas Gerais, Brazil; 5 Faculty of Medicine, Federal University of Minas Gerais, Belo Horizonte, Minas Gerais, Brazil; University of Melbourne, AUSTRALIA

## Abstract

The aim of this study was to develop and evaluate the effects of chitosan inserts for sustained release of the angiotensin-converting enzyme 2 (ACE2) activator, diminazene aceturate (DIZE), in experimental glaucoma. Monolayer DIZE loaded inserts (D+I) were prepared and characterized through swelling, attenuated total reflectance Fourier transformed infrared spectroscopy (ATR-FTIR), differential scanning calorimetry (DSC) and *in vitro* drug release. Functionally, the effects of D+I were tested in glaucomatous rats. Glaucoma was induced by weekly injections of hyaluronic acid (HA) into the anterior chamber and intraocular pressure (IOP) measurements were performed. Retinal ganglion cells (RGC) and optic nerve head cupping were evaluated in histological sections. Biodistribution of the drug was accessed by scintigraphic images and *ex vivo* radiation counting. We found that DIZE increased the swelling index of the inserts. Also, it was molecularly dispersed and interspersed in the polymeric matrix as a freebase. DIZE did not lose its chemical integrity and activity when loaded in the inserts. The functional evaluation demonstrated that D+I decreased the IOP and maintained the IOP lowered for up to one month (last week: 11.0±0.7 mmHg). This effect of D+I prevented the loss of RGC and degeneration of the optic nerve. No toxic effects in the eyes related to application of the inserts were observed. Moreover, biodistribution studies showed that D+I prolonged the retention of DIZE in the corneal site. We concluded that D+I provided sustained DIZE delivery *in vivo*, thereby evidencing the potential application of polymeric-based DIZE inserts for glaucoma management.

## Introduction

Glaucoma is an ocular disorder, with multi-factorial etiology, characterized by progressive optic nerve degeneration that results in visual field loss and irreversible blindness [1, 2]. More than 60 million people around the world are affected by glaucoma and it has been estimated that approximately 8 million people in the world suffer bilateral blindness caused by this disease [3]. The most well studied risk factor for glaucoma is increased intraocular pressure (IOP), which damages the optic nerve [4].

Eye drops are still the mainstay for glaucoma management, accounting for approximately 90% of the all ophthalmic treatments. Nevertheless, only 1% to 7% of the administered drugs actually reach the aqueous humor [5–7]. The inefficiency of this route is mainly attributed to the precorneal tear clearance mechanism, the highly selective anterior corneal epithelial barrier and the patient compliance, a factor that is quite unpredictable and difficult to control. Therefore, the development of new vehicles and drug formulations that enhance bioavailability and, consequently, reduce the number of administered doses requiring less patient efforts, represents an important aspect to control glaucoma progression [7].

Controlled drug delivery systems have been developed to overcome eye drops limitations [8]. Such systems can achieve prolonged therapeutic drug concentrations in ocular target tissues while limiting systemic exposure and side effects and improving patient adherence to therapy [9]. Non-implantable drug delivery devices, often named inserts, are placed in the fornix of the conjunctival sac of the lower eyelid, where they are exposed to tears. This route of drug delivery is used to treat conditions that affect the anterior segment of the eye [10, 11]. Inserts are often produced as a polymeric matrix made of degradable polymers. Chitosan seems to be a suitable polymeric matrix for ophthalmic inserts, as it is a non-toxic, biocompatible and biodegradable polymer [7, 12, 13].

The renin-angiotensin system (RAS) is well known for its role in regulation of blood pressure, electrolyte balance and vascular remodeling [14, 15]. The presence of precursors and enzymes which are necessary for angiotensin (Ang) II generation, the primary effector molecule of the RAS, in the eyes suggests that eyes possess a local RAS with physiological roles and pathological implications [16–20]. Among the new recognized components of the RAS, several studies have described the pathophysiological significance of the axis formed by angiotensin-converting enzyme (ACE) 2, Ang-(1–7) and Mas receptor [21–24]. Ang-(1–7) is synthesized mainly by ACE2 and interacts with the G-protein-coupled Mas receptor to exert its functions. This axis balances the vasoconstrictor and proliferative effects triggered by the activation of the ACE/Ang II/AT_1_ receptor branch of the RAS. Thus, it represents an endogenous counter regulatory pathway within the RAS [25]. Recently, we have reported that systemic and topical administration of the ACE2 activator, the compound diminazene aceturate (DIZE), prevented the elevation, as well as reduced the IOP of glaucomatous rats. These effects were mediated by Mas and involved neuroprotection of the retinal ganglion cells (RGC) and facilitation of the aqueous humor drainage [26].

Thus, in view of the fact that activation of endogenous ACE2 is a potential strategy to develop new antiglaucomatous agents, we elaborated a non-implantable drug delivery device made of chitosan and containing DIZE to test in glaucomatous rats. In other words, this study aimed to formulate, characterize and evaluate the *in vivo* activity against elevated IOP of a chitosan-based insert for sustained release of DIZE.

## Materials and Methods

### Material

Medium molecular weight chitosan and DIZE were supplied by Sigma-Aldrich (St. Louis, Mo, USA). Glacial acetic acid was purchased from Merck (Darmstadt, Germany). All other reagents were of analytical grade.

### Preparation of the inserts

Monolayer DIZE loaded inserts (D+I: DIZE + chitosan) were prepared by using solvent/casting technique, according to previous studies [27, 28]. First, a solution containing 4 mg/mL of DIZE and 15 μL/mL of acetic acid was prepared. Then, chitosan was added at the concentration of 20 mg/mL to obtain a viscous dispersion, which was magnetically stirred overnight to ensure homogeneity of both drug and polymer. The dispersion was casted, at room temperature, in circular silicone-molded trays (SMT) containing individual 5 mm × 2 mm wells [29]. After casting, inserts were gently removed from the SMT and stored in recipients protected from light and air humidity. Placebo inserts (P+I: chitosan only) were produced similarly, but changing the solution containing DIZE and acetic acid to another containing only acetic acid.

### Characterization of the inserts

#### Swelling studies

Swelling studies of the inserts were carried out in a phosphate buffer solution pH 7.4 (PBS). Each insert was weighed and placed in PBS for predetermined periods of time (5, 10, 20, 40, 60 and 90 min), as described by [30]. After immersion, the inserts were removed from the medium, the excess surface water was eliminated by using filter paper and the inserts were weighed. The degree of swelling was calculated by using the following equation [31]: Swelling index = [(*W*_*t*_-*W*_*0*_)/*W*_*0*_], where the weight of the swollen insert after predetermined period of time is represented by *W*_*t*_ and the original weight of the insert at zero time is represented by *W*_*0*_. This experiment was performed in triplicate (n = 3).

#### Attenuated total reflectance Fourier transformer infrared spectroscopy analysis

Attenuated total reflectance Fourier transformer infrared spectroscopy (ATR-FTIR) spectra were taken to confirm the chemical stability of DIZE after preparation of the inserts. ATR-FTIR spectra of DIZE inserts, placebo inserts and powdery DIZE (n = 3 in each group) were recorded on a Perkin Elmer FTIR spectrometer, Model Spectrum One (Perkin Elmer Instrument, USA). Spectra form 4000 to 600 cm^-1^ was recorded.

#### Differential scanning calorimetry analysis

Differential scanning calorimetry (DSC) measurements were carried out in a Shimadzu DSC50 (Shimadzu Corporation, Kyoto, Japan). Samples (D+I, P+I and DIZE, n = 3 in each group) were packed in an aluminum crucible and heated at a rate of 10°C/min. Nitrogen, at the rate of 20 mL/min, was used as a purge gas during the role analysis. The specimens were heated from -50°C to 200°C (RUN 1). Afterward, the specimens were cooled to -50°C at the same rate of 10°C/min and then they were reheated to 400°C at a rate of 10°C/min (RUN 2).

#### Scanning electron microscopy (SEM) analysis

The morphology of the inserts was evaluated using a JEOL (Akishima-Shi, TKY, Japan) scanning electron microscope, model JSM-6360LV, operating at 15 kV. The samples were prepared by freezing the inserts in liquid nitrogen. After freezing, the inserts were fractured. Next, the surface and sides of the inserts were analyzed. The devices were analyzed at suitable acceleration voltages using varying magnification for each sample. Representative electron micrographs were also taken (n = 3).

#### Quantification of DIZE in inserts

The amount of DIZE in the inserts was quantified by UV/Vis spectroscopy. A Shimadzu ultraviolet spectrometer (Shimadzu Corporation, Kyoto, Japan) was used at a wavelength of 425 nm. The method was validated in accordance with ICH guidelines (ICH, 1996). The DIZE concentration ranged from 4.0 to 20 μg/mL in 10% NaOH (w/v) (y = 0.03134x+ 0.00179, R^2^ = 0.9999; n = 5).

#### In vitro drug release

*In vitro* drug release was evaluated using the Franz cell system (n = 3) [32]. A cellulose acetate membrane with 0.45 μm pores was used to split the insert compartment from the receptor liquid compartment. PBS was used as a receptor liquid and the glass cells were incubated at 37 ± 0.5°C. At appropriate intervals, all the receptor liquid was withdrawn from the glass cells and an equal volume of the same receptor liquid was added to maintain a constant volume. The amount of the drug released was evaluated by UV/Vis spectrophotometry (Shimadzu ultraviolet spectrometer, Shimadzu Corporation, Kyoto, Japan). 10% NaOH (w/v) was added to all samples before quantification.

### Animals

Male Wistar rats weighing 180 to 220 g were obtained from the animal facility of the Faculty of Pharmacy, Federal University of Minas Gerais. The animals were housed in a temperature-controlled room (22–23°C) lit by fluorescent lights with a 12–12h light-dark cycle. Water and food were available *ad libitum*. The experimental protocols were performed in accordance with institutional guidelines approved by the Ethics Committee in Animal Experimentation of the Federal University of Minas Gerais, Brazil (CETEA-UFMG), which are in accordance with the National Institutes of Health (NIH) Guidelines for the Care and Use of Laboratory Animals (protocols #251/11 and #211/13). In addition, this study is conformed to the Association for Research in Vision and Ophthalmology (ARVO) statement for the use of animals in ophthalmic and vision research.

### Intraocular pressure evaluation

IOP measurements were performed using an applanation tonometer TonoPen XL (Mentor, Norwell, MA, USA) that was calibrated before use. To obtain the measures, unsedated animals were topical anesthetized by instillation of 0.4% benoxinate hydrochloride, and then were carefully contained with a small cloth. The tonometer was applied perpendicular to the more apical side of the cornea and three readings of IOP (with standard error up to 5%) were acquired in each eye. The average of these three measures was considered the corresponding value of IOP. IOP measurements were performed at the same time each day or week (between 11:00 AM and 12:00 PM) in order to avoid circadian IOP changes. The tonometrist was masked to the treatment and an assistant performed the randomization process. IOP was analyzed before surgery in order to obtain the baseline values and then weekly until the end of the experimental period.

### Systemic blood pressure measurement

The mean arterial pressure (MAP) was evaluated by a tail cuff method, which is a noninvasive computerized system for measuring blood pressure (Kent Scientific Corporation, Torrington, CT, USA). This tail-cuff blood pressure system utilizes volume pressure recording sensor technology to measure the rat tail blood pressure. The animals (n = 5 per group) were acclimated one day before the beginning of the experiments to restraint and to tail-cuff inflation. The restraint platform was maintained at approximately 32–34°C. For each session the rat was placed in an acrylic box restraint and the tail was involved by the compression cuff that measured the blood pressure 15 times, to calculate the average.

### Induction of glaucoma

Unilateral glaucoma was induced in the right eye by injection of 30 μL of hyaluronic acid (HA, BS Pharma) (10 mg/mL) into the anterior chamber through the clear cornea, near to the corneoscleral limbus using an hypodermic needle (22 gauge), once a week, for 6 weeks, in the same calendar day and time, according to Moreno and colleagues [33]. Rats were anesthetized intramuscularly with a mixture of ketamine (70 mg/kg) and xylazine (10 mg/kg). In addition, two drops of 0.4% benoxinate hydrochloride were instilled directly on the cornea as a local anesthetic. No procedures were performed in the contralateral eyes (non-glaucomatous eyes—control groups). Evaluation of IOP and MAP were carried out one day before the next HA injection.

### Experimental protocols and treatments

The animals (n = 5 in each group) were divided into six groups: (i) non-glaucomatous rats treated with saline (vehicle) (CTRL–untreated non-glaucomatous rats); (ii) non-glaucomatous rats treated with placebo inserts (CTRL + P+I–placebo non-glaucomatous rats); (iii) non-glaucomatous rats treated with DIZE inserts (CTRL + D+I–treated non-glaucomatous rats); (iv) glaucomatous rats treated with saline (vehicle) (GLAU–untreated glaucomatous rats); (v) glaucomatous rats treated with placebo inserts (GLAU + P+I–placebo glaucomatous rats); and (vi) glaucomatous rats treated with DIZE inserts (GLAU + D+I–treated glaucomatous rats). Only a single insert (1 mm x 2 mm) were placed in the fornix of the conjunctival sac of the lower eyelid after the establishment of ocular hypertension. In other words, the treatments initiated after confirmation of the elevated IOP, i.e. 1 week after the first injection of HA and lasted for 4 weeks. Importantly, the inserts are mucoadhesive, i.e. they possess the ability to adhere to the ocular mucosa. In order to guarantee the adhesion process, the inserts were hydrated before the application in the eye and, as the administration of the insert was performed immediately after the HA injection, the animals were anesthetized during the application. Thus, expulsion of the inserts was minimized.

In biodistribution studies (n = 6 in each group), the animals were divided into two groups: (i) non-glaucomatous rats treated with only one DIZE eye drop (DIZE eye drops) and (ii) non-glaucomatous rats treated with DIZE inserts (D+I).

### Histological analysis

After enucleation, two small sagittal sections were made in the nasal and temporal sides of the eyes (n = 7 in each group) and, immediately, they were immersed in Bouin’s fixative for approximately 24 hours. Thereafter, they were dehydrated in increasing concentrations of ethanol (70, 80, 90, 95 and 100%), diaphanized in xylene, and included and embedded in Paraplast. Semi-serial 6 μm-sections with 60 μm of interval between each section were obtained using a microtome (model HM335E, Microm, Minnesota, USA). For histological analysis and RGC counting, histological sections were stained with hematoxylin and eosin (HE). The RGC counting was performed in 10 histological slides from each eye sample covering the whole extension of the retina, including the area of the optic nerve, using a microscope (Olympus BX 41, Irving, TX).

### Biodistribution studies

Biodistribution studies were conducted by *ex vivo* and *in vivo* (scintigraphic imaging) approaches. They were based on free and entrapped DIZE radiolabeled with technetium-99m (^99m^Tc). DIZE was dissolved in water (1 mg/mL) and radiolabeled with ^99m^Tc by a direct labeling method using stannous chloride as reducing agent. After 15 minutes, this radiolabeled drug solution was used to prepare DIZE inserts (^99m^Tc-D+I). ^99m^Tc-DIZE eye drops were used as control. For the scintigraphic imaging study, samples were prepared and radiolabeled to obtain enough activity to produce and acquire the images. The animals (n = 6 in each group) were anesthetized intramuscularly with a mixture of ketamine (70 mg/kg) and xylazine (10 mg/kg) and placed on a table in prone position under a gamma camera (Mediso, Hungary) employing a low-energy high-resolution collimator (LEHR). Images were acquired using a 256x256x16 matrix size with a 20% energy window set at 140 keV for a period of 10 min. Images were obtained at 30 min, 2, 4, 6 and 18 h after topical ocular administration of 7.4 MBq (200μCi) of both radiolabeled formulations. After the 18 h-images, blood samples were collected by subclavian artery puncture from anesthetized animals. The rats were euthanized by cervical dislocation and the spleen, heart, liver, kidneys, stomach, small and large intestines and eyes were collected and weighed. Determination of radioactivity in the organs was achieved through an automatic gamma counter (Wizard, Finland). The readings were conducted within the 130–150 keV energy window for 1 min. An aliquot of 99mTc-DIZE containing the same injected dose was counted simultaneously in a separate tube, which was defined as 100% radioactivity. The results were expressed as the percentage of injected dose/g of tissue (% injected dose/g).

### Statistical analysis

All data were expressed as mean ± SEM. The results regarding the effects of DIZE on IOP, as well as morphometric analysis of RGC were analyzed using One-way ANOVA followed by the Newman-Keuls post-test. Swelling index and MAP were analyzed using two-way ANOVA followed by the Bonferroni post-test. Comparative results of the biodistribution study were analyzed using unpaired Student’s t-test. All these tests were performed utilizing the GraphPad Prism 5 software. The significance level considered was p<0.05.

## Results

### Characterization of the inserts and *in vitro* drug release

**Fig 1:**
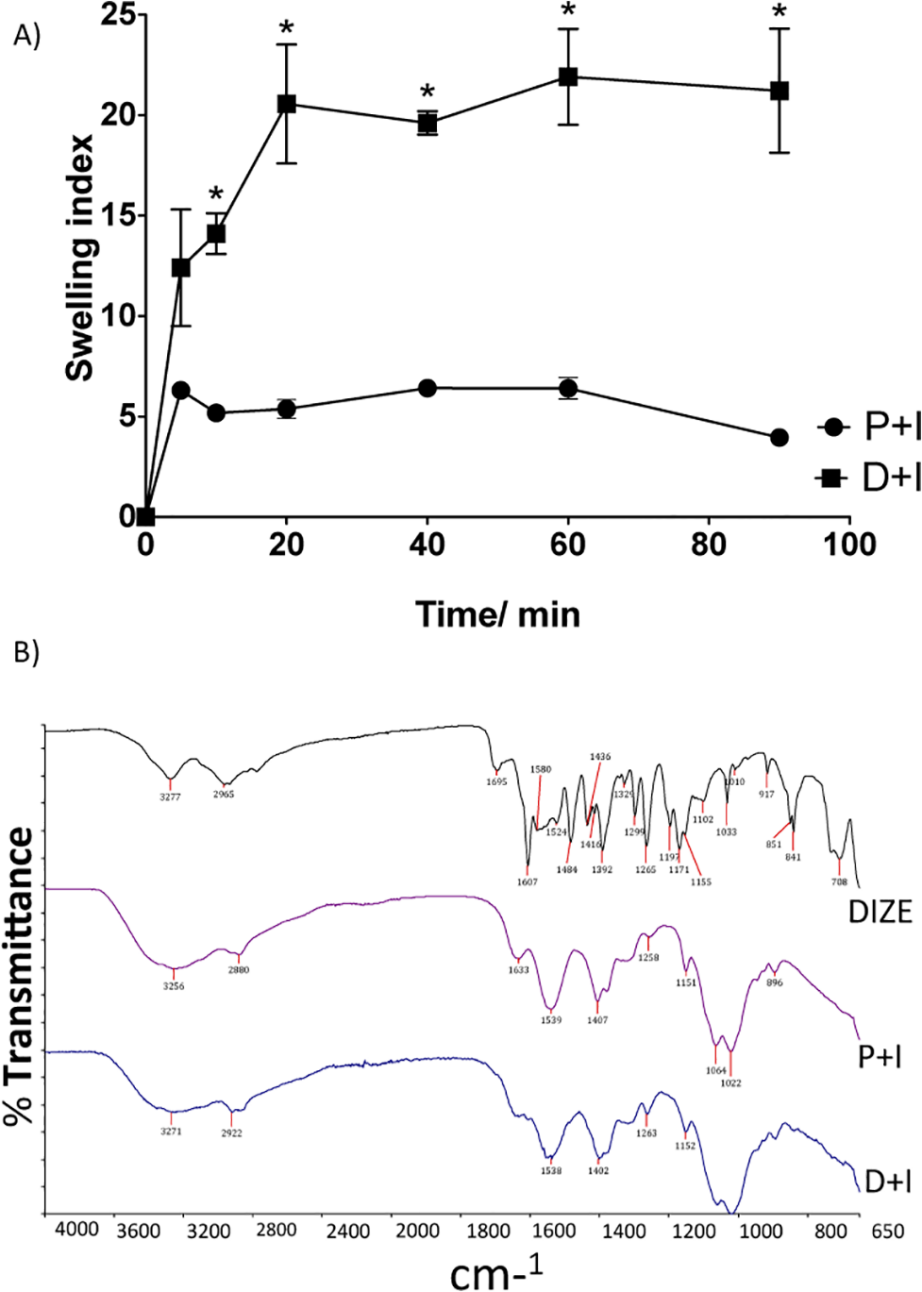
Characterization of chitosan inserts containing DIZE by swelling analysis and ATR-FTIR spectra. **(A)** Swelling index of placebo inserts (P+I: chitosan only) and DIZE inserts (D+I: Dize + chitosan) in PBS; n = 3 per group. D+I swelled more quickly and reached higher swelling indexes than P+I (*p < 0.001). Two-way ANOVA followed by the Bonferroni post test. **(B)** ATR-FTIR spectra of powdery DIZE (DIZE), P+I (chitosan only) and D+I (Dize + chitosan). D+I spectra showed characteristic bands both of P+I and DIZE. No new bands were identified in D+I.

Inserts were obtained as circular flexible membranes. The results of the swelling studies are shown in **Fig 1A**. Both placebo inserts and DIZE inserts were hydrated very quickly reaching more than 80% hydration in the first 20 min of water immersion. Nevertheless, the inserts did not break apart after the swelling process. It is possible to observe that DIZE increased the water uptake in the inserts. ATR-FTIR spectroscopy (**Fig 1B**) showed characteristic absorption bands of chitosan C = O stretching (amide I band) and overlapping of N-H (amine) vibration and N-H vibration (amide II) at 1633 and 1539 cm-1, respectively, in the P+I spectrum. These bands were preserved in the D+I spectrum without shifting. Moreover, characteristic absorption bands of DIZE were preserved without shifting (**Fig 1B**). On the other hand, the first band of the DIZE insert was widened and had a significant shifted to higher frequency (from 3256 to 3271 cm-1) when compared to placebo insert. Also, typical absorption bands of protonated groups of DIZE salt were identified at 1607 and 1484 cm-1 in DIZE spectrum but not in D+I spectrum. Despite these differences among the spectra, it is important to notice that FTIR of D+I did not show any new band (**Fig 1B**), indicating that DIZE did not chemically react with the polymeric matrix. DIZE was entrapped as a freebase and interacted with the polymeric matrix through hydrogen bonding.

**Fig 2:**
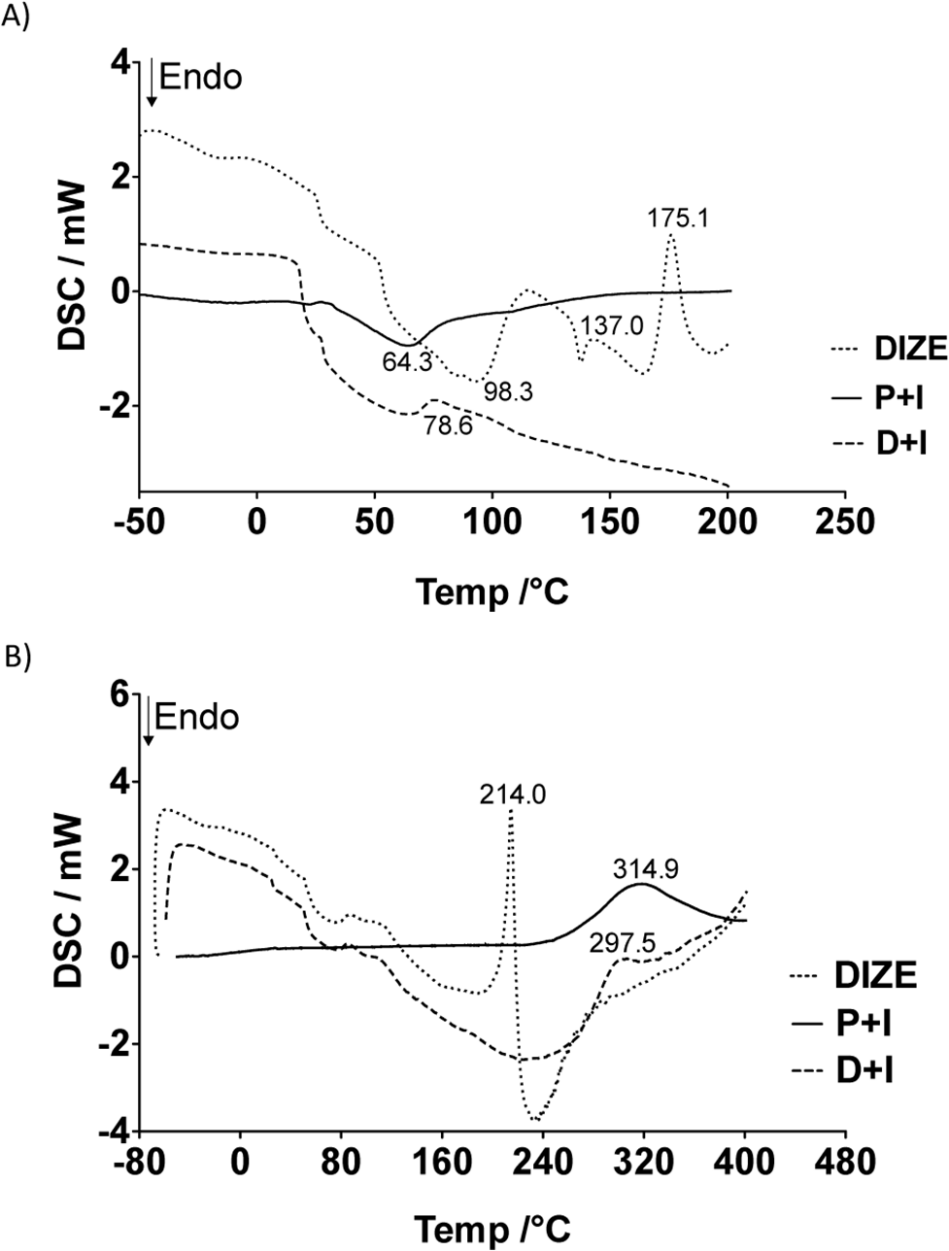
Differential scanning calorimetry (DSC) curves of DIZE, placebo inserts (P+I) and DIZE inserts (D+I). Peaks at 64.3°C **(A)** and 314.9°C **(B)** on P+I curve are attributed to evaporation of residual water and degradation of the main polymeric chain, respectively. On DIZE curves, peaks at 98.3°C **(A)** and 137.0°C **(B)** are attributed to loss of water from the salt and peaks at 175.1°C **(A)** and 214.0°C **(B)** indicates the decomposition of DIZE. Peaks of decomposition of DIZE were not detected in D+I curves; n = 3 per group.

DSC analysis revealed that P+I presented a broad endothermic peak at 64.3°C **(Fig 2A)**, as well as a broad exothermic peak at 314.9°C **(Fig 2B)** on the first and second scan curves, respectively. Both peaks were irregular and can be attributed to an evaporation of residual water and a degradation of the main polymeric chain, respectively. DIZE curves showed a long endothermic phase with maximum at 98.3°C and an endothermic peak at 137.0°C, which are related to the loss of water from the salt **(Fig 2A)**. At 175.1°C, an exothermic peak, indicating the first decomposition of DIZE, was detected. At 214.0°C, a sharp exothermic event was detectable, which can be explained by a second phase of decomposition of the drug **(Fig 2B)**. Peaks of decomposition of DIZE were not detected in D+I curves. Moreover, it was observed a decrease in the temperature of chitosan degradation (from 314.9°C to 297.5°C) when DIZE was added to the inserts **(Fig 2A and 2B)**.

**Fig 3:**
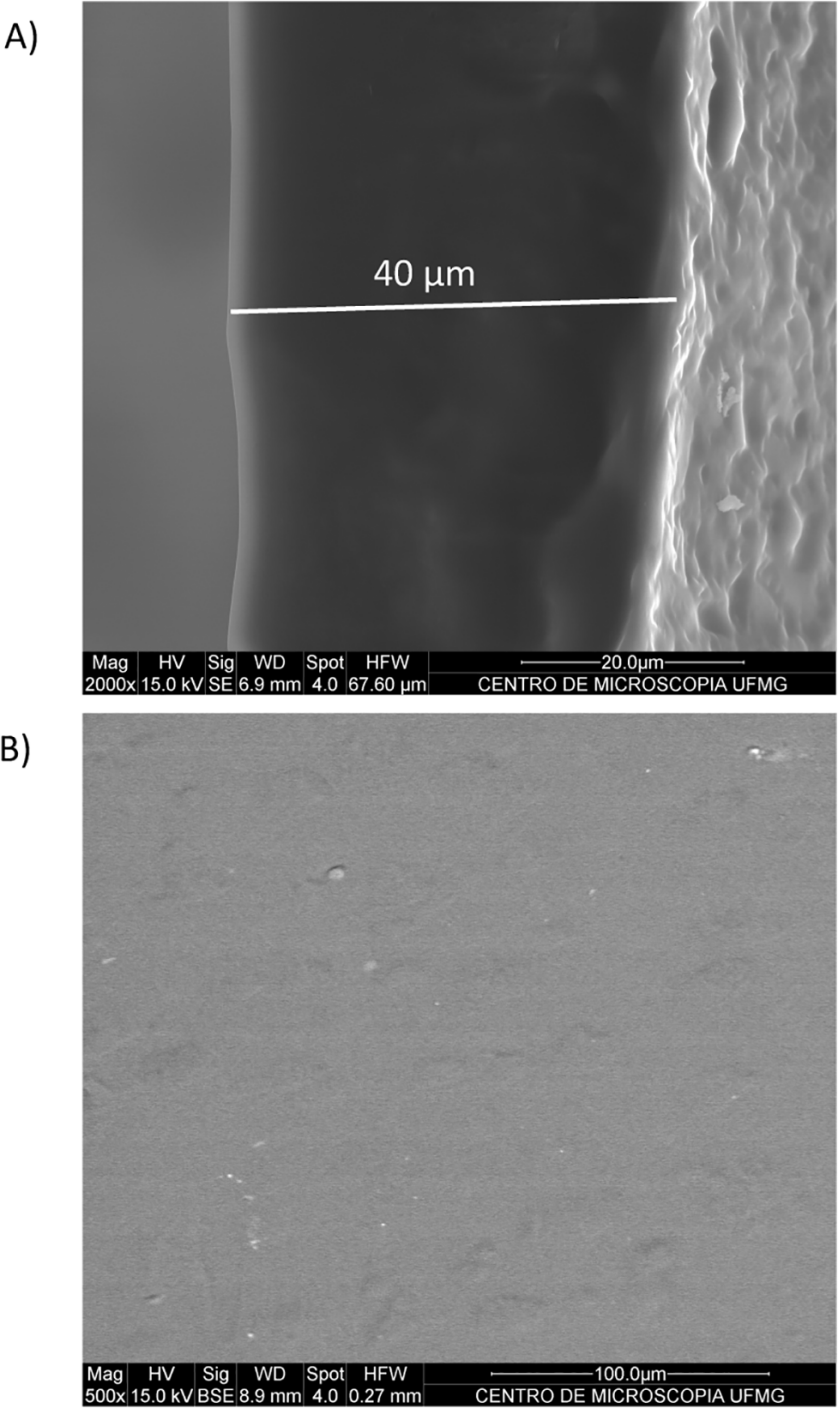
Representative scanning electron micrograph of DIZE inserts (D+I). **(A)** Micrograph of the lateral side of the insert (n = 3). **(B)** Micrograph of the surface of the insert (n = 3). D+I are uniform without granular particles in the middle or in the surface and measuring approximately 40 μm of thickness.

Morphological characterization of the inserts was performed using SEM, whose pictures are presented in **Fig 3**. D+I inserts are uniform without granular particles in the middle or in the surface and measuring approximately 40 μm of thickness.

**Fig 4:**
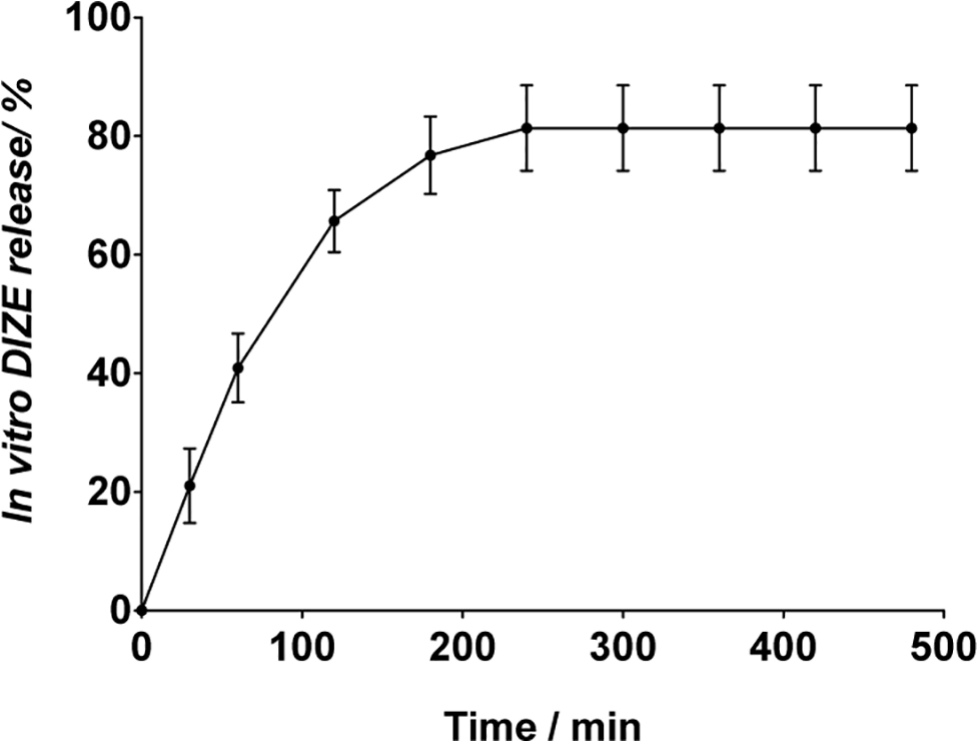
*In vitro* release of DIZE. In vitro release of DIZE from DIZE inserts (D+I) showing that approximately 80% of the drug was controlled released in four hours (n = 3).

The release of DIZE from D+I was monitored *in vitro*. Under physiological conditions of pH, it is possible to observe that approximately 80% of DIZE was controlled released during the first four hours. Interestingly, no further release was detected after this period **(Fig 4)**.

### Effects of the controlled release of DIZE on IOP of glaucomatous rats

**Fig 5:**
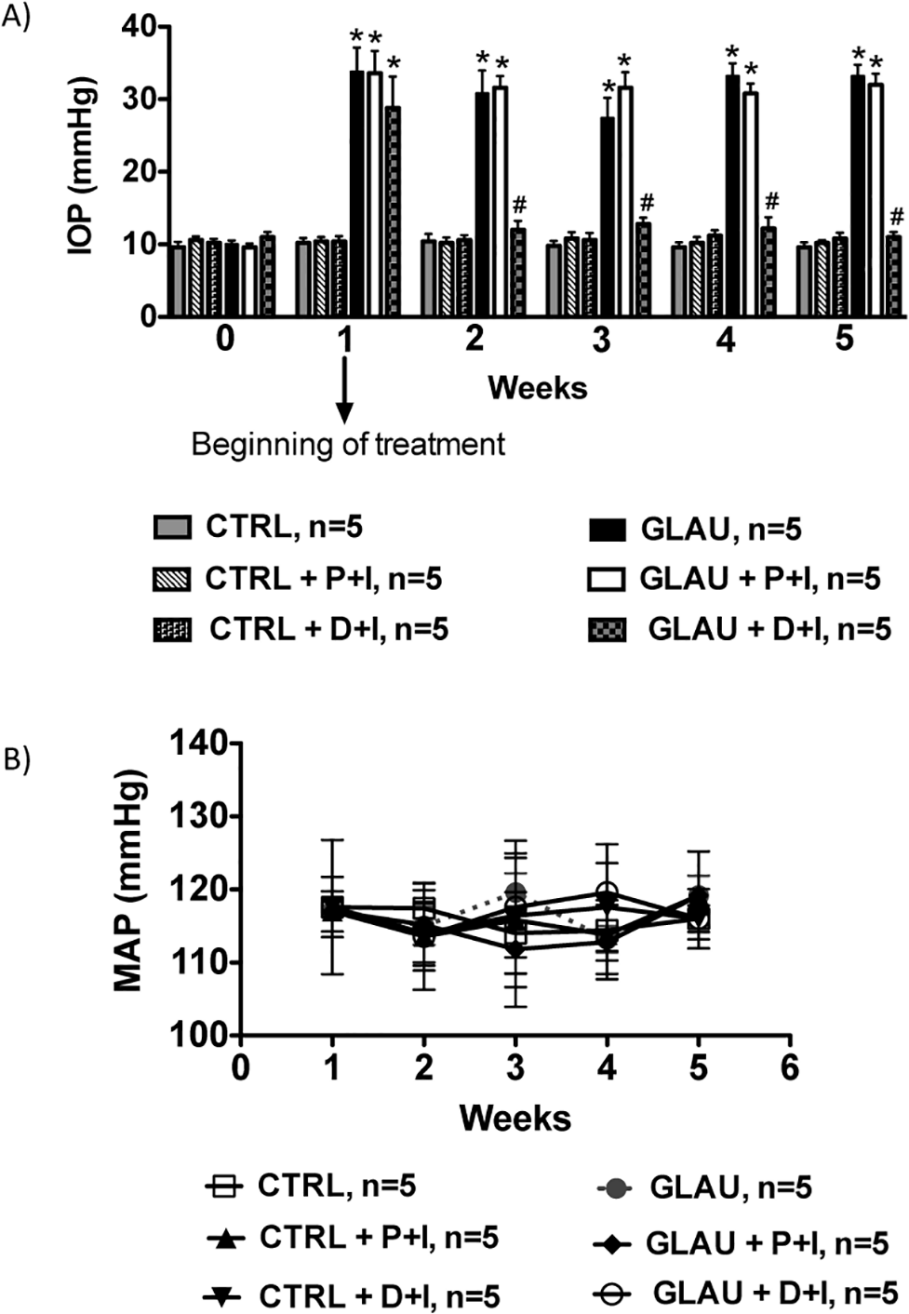
Effects of DIZE inserts (D+I) on intraocular pressure (IOP) and mean arterial pressure (MAP). **(A)** It is possible to observe that even after the establishment of ocular hypertension, DIZE was able to reduce the IOP returning it to baseline values; n = 5 per group. **(B)** The antiglaucomatous effects of D+I did not interfere in the MAP; n = 5 per group. *p<0.05 vs. control (CTRL) and #p<0.05 vs. glaucoma (GLAU) (One-way ANOVA followed by the Newman-Keuls post test).

Functionally, the biological efficiency of our inserts to release DIZE was tested in an experimental model of glaucoma induced by intraocular injection of HA. **Fig 5A** shows the IOP of all experimental groups during the period of 6 weeks. The IOP of control glaucomatous animals (33.2 ± 1.6 mmHg at the last week) and of glaucomatous animals treated with P+I (31.8 ± 1.5 mmHg at the last week) were significantly higher than control animals treated or not with P+I (control non-treated rats at the last week: 9.6 ± 0.67 mmHg and control rats treated with placebo at the last week: 10.4 ± 0.6 mmHg). After one week of the treatment with D+I, the IOP of glaucomatous animals lowered at the levels of control animals (last week: 11.0 ± 0.7 mmHg). Importantly, this effect lasted for the following four weeks of evaluation. No significant changes in IOP were observed in non-glaucomatous animals treated with inserts containing DIZE. Of note, the anti-glaucomatous effects of the inserts containing DIZE were not accompanied by alterations in MAP **(Fig 5B)**.

### Effects of the controlled release of DIZE on RGC of glaucomatous rats

**Fig 6:**
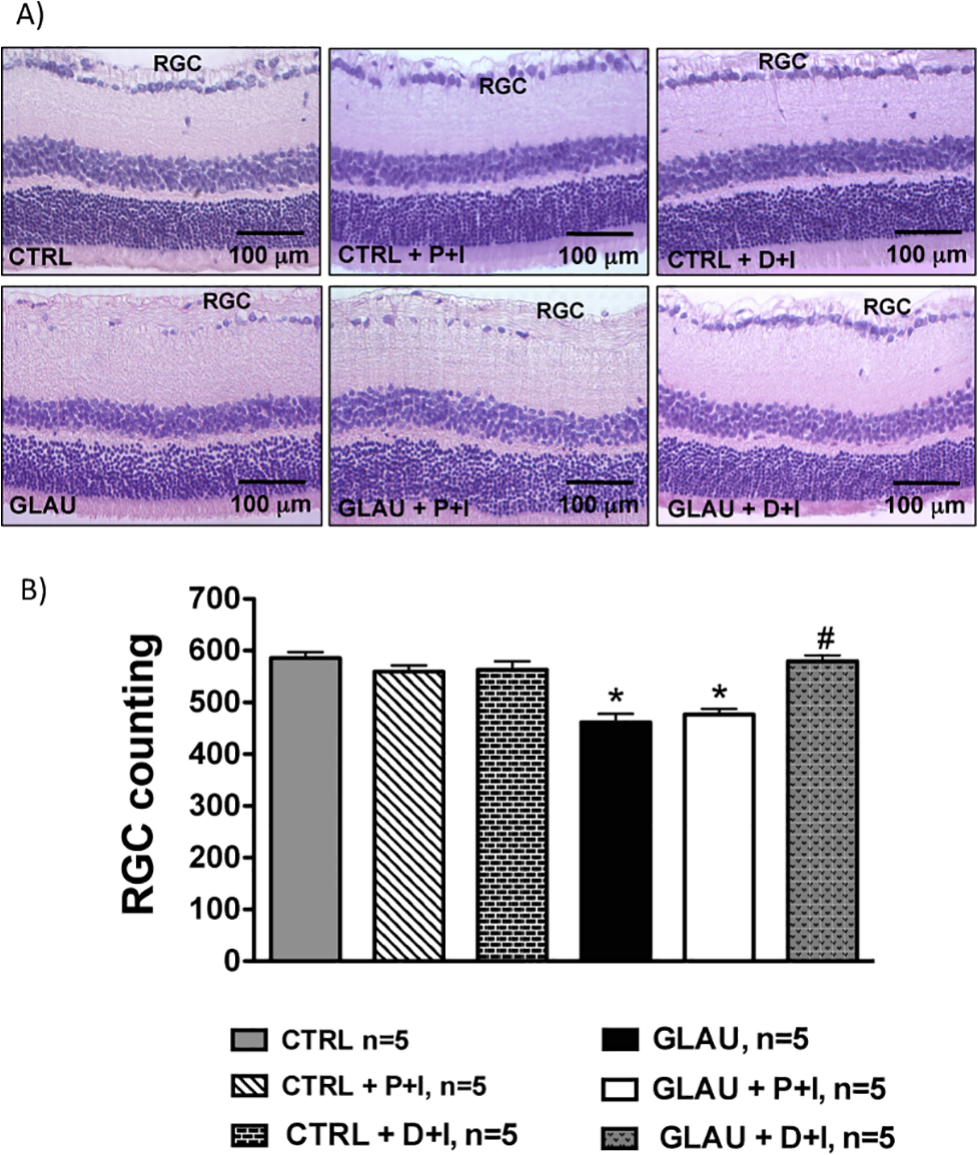
Histological analysis of retinal ganglion cells (RGC). **(A)** Representative photomicrographs of retinas showing the smaller number of RCG in control glaucomatous rats treated or not with placebo inserts and the beneficial effects of DIZE inserts in these cells; n = 5 per group. **(B)** Quantification of RGC in retinas of rats; n = 5 per group. *p<0.05 vs. control (CTRL) and #p<0.05 vs. glaucoma (GLAU) (One-way ANOVA followed by the Newman-Keuls post test). CTRL: control non-treated group; CTRL+P+I: control group that received placebo inserts; CTRL+D+I: control group that received DIZE inserts; GLAU: glaucoma non-treated group; GLAU+P+I: glaucoma group that received placebo inserts; and GLAU+D+I: glaucoma group that received DIZE inserts.

The IOP lowering effects of DIZE were followed by preservation of RGC. As seen in **Fig 6**, we found that control glaucomatous animals and glaucomatous rats treated with P+I presented a large reduction in the number of RGC (glaucomatous animals—GLAU: 461.6 ± 16.6 cells and glaucomatous rats + placebo inserts—GLAU+P+I: 476.4 ± 11.0 cells). This effect was completely restored by the inserts containing DIZE (non-glaucomatous animals—CTRL: 595.3 ± 11.7 cells vs. glaucomatous rats + DIZE inserts—GLAU+D+I: 589.7 ± 10.9 cells). Again, no significant changes were induced by D+I in non-glaucomatous animals.

**Fig 7:**
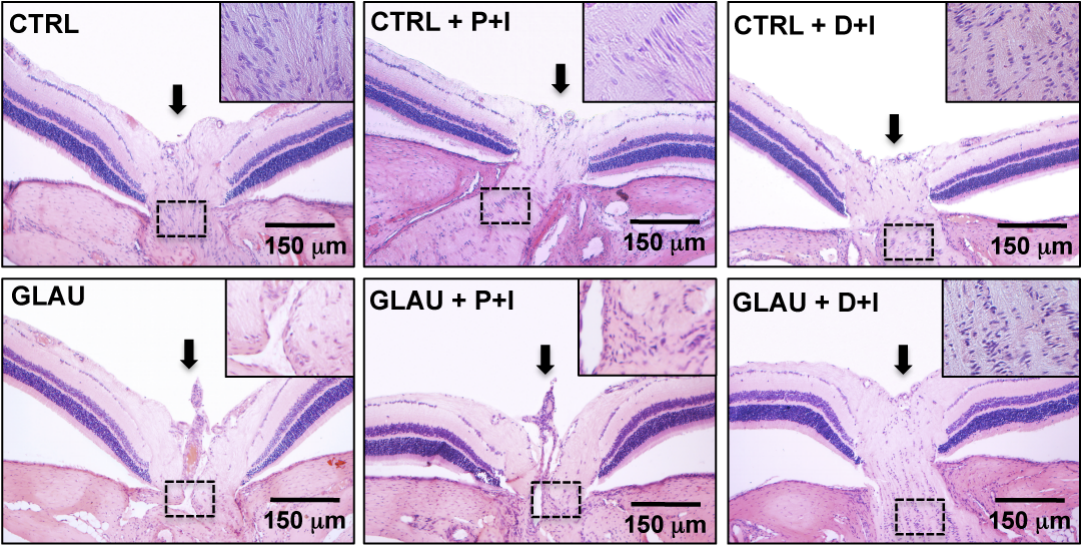
DIZE inserts (D+I) promoted neuroprotection in retinas of glaucomatous rats. Representative photomicrographs of excavation of the optic nerve (arrows). Note the optic nerve head cupping in control glaucomatous animals treated or not with placebo inserts (P+I) when compared to all other groups; n = 5 per group. Treatment with D+I was able to reverse this effect. In detail are representative photomicrographs of longitudinal sections of the optic nerve. A large reduction in the neural fibers was observed in control glaucomatous rats treated or not with P+I. CTRL: control non-treated group; CTRL+P+I: control group that received placebo inserts; CTRL+D+I: control group that received DIZE inserts; GLAU: glaucoma non-treated group; GLAU+P+I: glaucoma group that received placebo inserts; and GLAU+D+I: glaucoma group that received DIZE inserts.

Additionally, the reduction in the number of RGC caused by elevated IOP was accompanied by a severe loss of neural fibers with consequent increase in the optic nerve head cupping. These effects were abolished by the treatment with inserts containing DIZE **(Fig 7)**. Altogether, these data indicate that controlled release of DIZE induced a neuroprotection by decreasing the loss of RGC and neural fibers.

### Biodistribution studies

**Fig 8:**
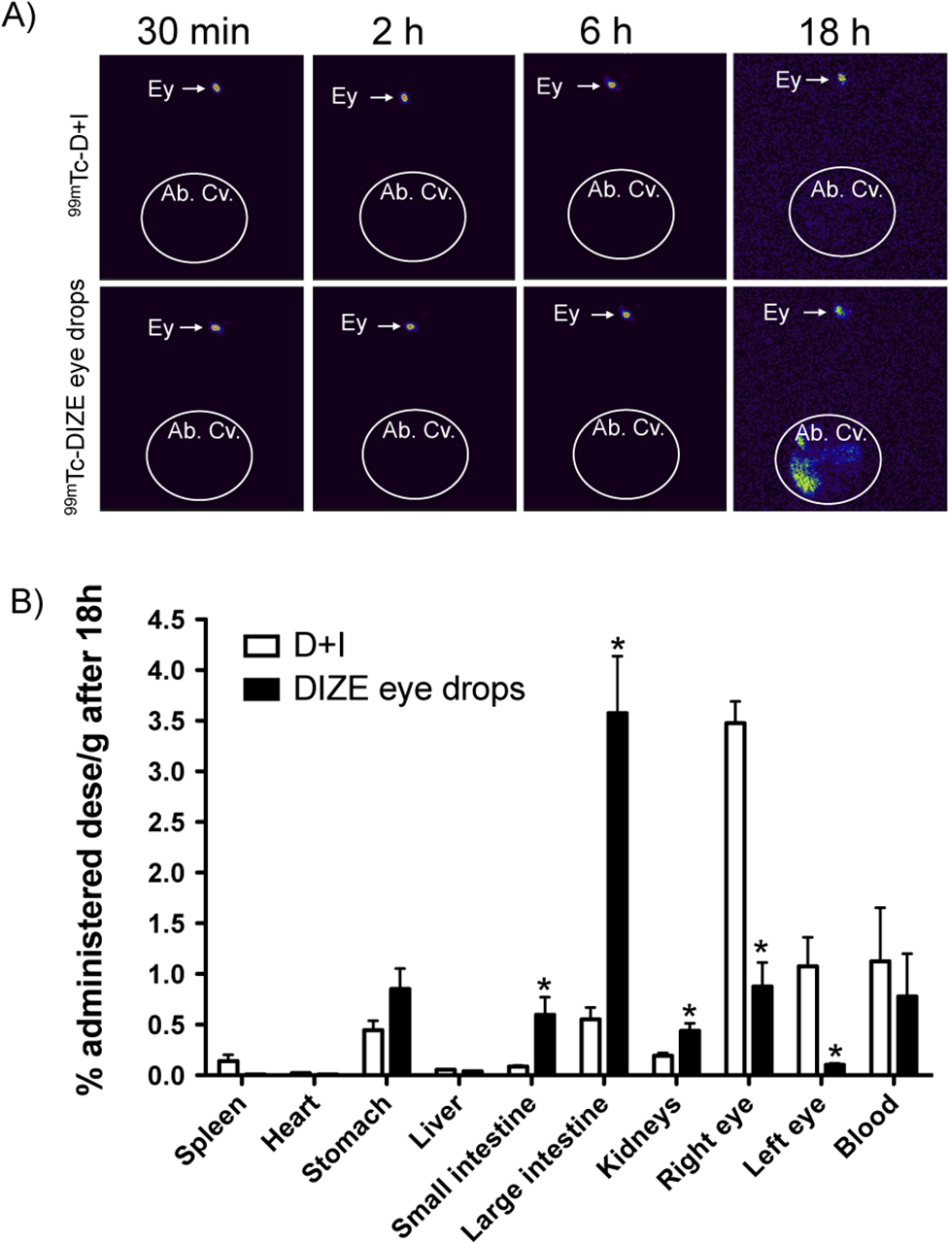
*In vivo* drug biodistribution. **(A)** Representative images after 30 min, 2, 4, 6 and 18 hours of administration of the radiolabeled formulations; n = 5 per group. Note that ^99m^Tc-DIZE eye drops began to clear from the corneal region reaching the gastrointestinal tract via the nasolacrimal drainage system after 18 hours; while ^99m^Tc-D+I remained in the eye. Ey: eye; Ab. Cv.: abdominal cavity. **(B)** Percentage of administered dose per gram after 18 hours in spleen, heart, stomach, liver, small intestine, large intestine, kidneys, right eye, left eye and blood. *p<0.05 vs. the same organ from rats treated with ^99m^Tc-D+I (Unpaired Student’s *t*-test).

Radiolabeling of DIZE with ^99m^Tc showed a radiochemical purity of 81.39% ±0.51%. It was observed that the amount of ^99m^Tc-DIZE that began to clear from the corneal region reaching the gastrointestinal tract via the nasolacrimal drainage system was significantly higher in animals treated with ^99m^Tc-DIZE eye drops than in those rats treated with ^99m^Tc-D+I **(Fig 8A)**. Quantitatively, the animals treated with ^99m^Tc-DIZE eye drops showed approximately 6.8 times more drug in the small intestine (0.59 arbitrary units) than animals of the ^99m^Tc-D+I group (0.08 arbitrary units). In the large intestine, ^99m^Tc-DIZE eye drops rats presented 6.4 times more drug than ^99m^Tc-D+I animals (3.57 arbitrary units vs. 0.55 arbitrary units, respectively). Also, the amount of drug in the kidneys of ^99m^Tc-DIZE eye drops group was 2.3 times higher than in ^99m^Tc-D+I group (0.44 arbitrary units vs. 0.19 arbitrary units, respectively). In contrast, ^99m^Tc-D+I animals presented a significantly higher amount of drug in the right and left eyes compared to ^99m^Tc-DIZE eye drops animals after 18 hours **(Fig 8B)**. Together, these results suggest that ^99m^Tc-D+I prolonged the retention of DIZE at the corneal site, since the amount of drug that remained in the eye was significantly higher in this group. Also, it reduced the extent of nasolacrimal drainage, as the amount of ^99m^Tc-D+I was significantly lower in the gastrointestinal tract (small and large intestines) and kidneys of animals treated with ^99m^Tc-D+I.

## Discussion

Glaucoma is a chronic pathological condition that requires long-term treatment in order to stop the disease progression and to avoid the irreversible blindness. In many glaucomatous patients, medical therapy consists of eye drops [28], nevertheless, controlled drug delivery systems offer manifold advantages over conventional systems [34]. In this context, this study was designed to develop and characterize a new drug delivery system, a chitosan-based device containing an activator of intrinsic ACE2 (DIZE), for sustained release in eyes. Moreover, we evaluated the effects of this device in experimental glaucoma. Our main findings were that topical inserts loaded with DIZE were very efficient as a controlled drug delivery system, lowering the IOP by over a month after a single application and promoting neuroprotection by preserving RGC.

In agreement with a previous study, we found that DIZE increased the water uptake of the inserts [35]. Indeed, disorganization of the polymeric matrix can facilitate the water penetration [36]. Of note, the devices did not break apart and maintained its integrity even after the whole swelling process. Analyses of the ATR-FTIR spectra suggested that DIZE interacted with the polymeric matrix through hydrogen bonding. The absorption bands observed in D+I spectra were originally present either in DIZE and P+I spectra. This finding indicates that there was no obvious chemical reaction between the drug and matrix and that the drug did not lose its activity when loaded in the inserts. In contrast, typical absorption bands of protonated groups of DIZE salt were not preserved in D+I spectra. These bands were probably replaced by another band near to 1595 cm^-1^ overlapped by amide II and N-H vibration band [37]. This can be attributed to N-H vibration of DIZE freebase. Thus, it is possible to suggest that, although DIZE was used as a salt, it was entrapped in the insert as a freebase drug. Also, the first band of the insert spectra was significantly shifted to higher frequency (from 3256 to 3271 cm^-1^) and widened, suggesting the formation of hydrogen bonding between the drug and polymeric matrix. DSC curves also indicated that the drug interacted with the polymeric matrix, lowering the degradation temperature of the main polymeric chain. Also, the peaks of decomposition of DIZE were not detected in DSC curves of D+I, therefore indicating that the drug was molecularly dispersed in the matrix. This data corroborated the SEM results, which showed no granular particles in the middle or in the surface of the inserts. Again, these findings suggest that the drug was molecularly dispersed in the polymeric matrix, as predicted by DSC analysis. Considering the sustained release profile, our results are in keeping with findings reported by Soliman and Winnik (2008) who entrapped DIZE in carboxymethyldextran-PEG block micelles [38]. *In vitro* release of DIZE showed that approximately 80% of the drug was controlled released in four hours. Likely, the fast *in vitro* drug release was due to the constant hydration in the Franz cell system, promoting a constant and fast release of the drug by swelling. In the eye, such condition does not happen because the volume of liquid is limited (tears). Then, in this case, the drug will be released more slowly than *in vitro*.

To test the therapeutic efficacy of D+I in lowering IOP, we placed the inserts in the fornix of the conjunctival sac of rats with ocular hypertension. In all experiments, the inserts were well tolerated. No anterior chamber inflammation or corneal changes were observed after fluorescein administration in the eyes of the rats. After one week of application, insert matrix was completely mixed to the ocular mucosa. The device was able to lower IOP and completely restore the baseline values of IOP. Importantly, control glaucomatous eyes which received placebo inserts (GLAU+P+I) did not show any significant change in the elevated IOP. These *in vivo* data demonstrated that D+I reduced the IOP for up to 30 days and they were well tolerated by the animals. Of note, each chitosan insert was loaded with low amount of DIZE (200 μg) and it was effective for all experimental period. This feature is a tremendous advantage of any controlled drug delivery system because this avoids drug loss to systemic circulation since eye drops might lead to drug wastage and potential side effects [34]. Furthermore, eye drops are susceptible to rapid tear turnover resulting in low corneal bioavailability and rapid clearance. In this way, eye drops require frequent instillation with large drug loadings to maintain the drug concentration within the therapeutic window [39]. Noteworthy, the ocular hypotensive effect promoted by D+I did not interfere in the MAP. This characteristic is desirable for any antiglaucomatous drug or formulation as it increases its specificity reducing potential side effects.

The pathophysiological rationality to explore modulators of the RAS as antiglaucomatous agents come from previous studies. For instance, the effects of blockers of the ACE/Ang II/AT_1_ receptor axis on IOP have been evaluated in animals [40–42] and patients with glaucoma [43–45]. Recently, we have proposed activation of ACE2 as a new strategy to develop drugs to treat glaucoma since this approach increases the inactivation of Ang II and production of Ang-(1–7) [26]. Indeed, our present findings showed that increased activity of endogenous ACE2 promoted by ocular inserts containing DIZE reduced the IOP of glaucomatous rats. Interestingly, activation of ACE2 also induces beneficial effects on uveitis [46]. Thus, these findings, in addition to data reporting the expression of ACE2 in both human [47] and rodent retinas [26, 48] and the effectiveness of DIZE as an activator of ACE2 [49], are the rational to propose activation of intrinsic ACE2 as a new strategy to treat ocular diseases.

Although we did not investigate the molecular pathways underlying the effects of DIZE, possible mechanisms such as release of nitric oxide (NO) and prostaglandins induced by Ang-(1–7), suppressing inflammation and inhibiting cell proliferation may be involved [46, 50–52]. NO is a physiological active molecule present in rods, bipolar cells, amacrine cells and ganglion cells in retinas [53]. It promotes relaxation of trabecular meshwork cells and ciliary muscle and its production appears to be reduced in the context of primary open-angle glaucoma [50]. Additionally, treatment with indomethacin, an inhibitor of prostaglandins synthesis, abolished the IOP-lowering effect caused by enalaprilat, indicating that prostaglandins may mediate, at least in part, the ocular hypotensive effect of enalaprilat [40]. Indeed, it is well known that Ang II directly induces cell proliferation and contributes to the inflammatory process by increasing the expression of pro-inflammatory cytokines, chemokines and cell adhesion molecules via AT_1_ receptor [54]. Moreover, treatment with DIZE decreased the infiltration of inflammatory cells in both anterior and posterior segment and decreased the expression of inflammatory cytokines [46]. Thus, it is possible that one or more of these pathways may be related to the action of DIZE in IOP lowering effect. It should be observed that the trauma associated with the cannulation of the anterior chamber in our model can lead to activation of an inflammatory response, which can contribute to the increased IOP. Also, the large amount of HA may itself contribute to the inflammation in the anterior chamber. Therefore, at the moment, we can not rule out the possibility that the beneficial effect of DIZE was due to acting in the inflammatory process associated to the model and future studies should be conducted in other animal models of chronic IOP elevation for further understanding the underlying mechanisms involved in the DIZE IOP lowering effect.

It is worthy of note that DIZE was able to produce a reduction in IOP much greater than any commercially available compound. This finding may be related to the fact that ACE2 plays a double role in the RAS, i.e. it degrades Ang II and generates Ang-(1–7) [22]. Ang II increases the IOP of normal rats [44] and decreases the drainage of aqueous humor in primates [55] and in rabbits [56]. Also, it has been demonstrated the presence of polymorphisms of the Ang II type 1 receptor gene in patients with primary open angle glaucoma [57]. On the other hand, Ang-(1–7) is a heptapeptide that promotes vasodilation and antihypertensive effects which are counterregulatory actions to the Ang II effects [22]. Thus, activation of ACE2 might reduce IOP through both degrading Ang II and forming Ang-(1–7). Furthermore, increased ACE2 action and expression is observed when ACE2 activators are administered [24, 58]. This strongly suggests that these compounds induce their beneficial effects not only by forming Ang-(1–7) and/or degrading Ang II, but also through an unidentified mechanism that is able to increase the expression of ACE2. Drugs that promote IOP lowering effects act reducing the production of aqueous humor or increasing the drainage thereof [59]. In this context, we can not rule out the possibility that drugs able to modulate the RAS can act in this two ways. In a previous study, we demonstrated that DIZE increased the drainage of aqueous humor [26] and it has been suggested that captopril, an ACE inhibitor, reduces the aqueous humor production [44]. ACE inhibitors appear to promote synthesis of prostaglandins, increases in bradykinin levels, NO release and reduction in the formation of the vasoconstrictor peptide endothelin-1, which could consequently reduce IOP [60]. Thus, it is possible that one or more of these factors may contribute to the profound IOP reduction induced by DIZE.

Neuroprotective mechanisms also appear to be involved in the effects of D+I on glaucoma. We found characteristic optic nerve head cupping, death of RGC and axonal loss in control glaucomatous animals and in glaucomatous rats treated with placebo inserts. The D+I were able to decrease the death of RGC, as well as the optic nerve head cupping. Nowadays, neuroprotective agents are under extensive investigation since almost all drug therapies for treatment of glaucoma are based on lowering IOP or preventing the increase of IOP [26, 59]. Thus, classical agents often did not avoid RGC death leading to progressive vision loss. Consequently, there is a great interest in developing agents that not only reduce IOP but also promote neuroprotection.

Biodistribution studies evidenced that chitosan was able to enhance precorneal retention time of DIZE in the D+I group, likely due to mucoadhesive proprieties of the polymeric matrix. It has been already proved that chitosan shows a prolonged precorneal residence when delivered in the eye [61], leading to an increase in the bioavailability of the drug instilled. Here, we demonstrated that chitosan was able to afford this propriety to DIZE, which is probably because of the physicochemical interaction between the drug and polymeric matrix, as show in the characterization studies. Thus, the retention of the formulation delays its washout and results in an enhanced bioavailability of the drug.

One may argue that the mechanism of injury in our model involves a much larger ischemic component as compared with other experimental models. However, against this possibility, Moreno et al. (2005) have shown that eye’s general architecture was unaffected by injection of hyaluronic acid. Also, the histopathologic examination indicated no modification in the degree of chamber angle constriction in this model [33].

In the present study, we produced and properly characterized a drug delivery system able to enhance the ocular bioavailability of the novel ACE2 activator DIZE. As a result of its enhanced bioavailability, D+I treatment reduced the IOP for up to 4 weeks after a single application, resulting in neuroprotective effects in RGC. Additionally, our results successfully confirmed the advantages of delivering ophthalmic drugs through topical inserts, indicating that this approach is a potential therapeutic strategy to controlled release of antiglaucomatous ophthalmic drugs.
